# Folk Moral Objectivism: The Case of Harmful Actions

**DOI:** 10.3389/fpsyg.2021.638515

**Published:** 2021-07-28

**Authors:** Paulo Sousa, Aurélien Allard, Jared Piazza, Geoffrey P. Goodwin

**Affiliations:** ^1^Institute of Cognition and Culture, Queen's University Belfast, Belfast, United Kingdom; ^2^Institute Ethics History Humanities, University of Geneva, Geneva, Switzerland; ^3^Department of Psychology, Lancaster University, Lancaster, United Kingdom; ^4^Department of Psychology, University of Pennsylvania, Philadelphia, PA, United States

**Keywords:** folk meta-ethics, moral beliefs, objectivism, universalism, harm, injustice

## Abstract

It is controversial whether ordinary people regard beliefs about the wrongness of harmful actions as objectively correct. Our deflationary hypothesis, consistent with much of the evidence, is that people are objectivists about harmful actions *that are perceived to involve injustice*: when two parties disagree about whether such an action is wrong, people think that only one party is correct (the party believing that the action is wrong). However, Sarkissian and colleagues claimed that this evidence is misleading, showing that when the two disagreeing parties are from radically different cultures or species, people tend to think that both parties are correct (a non-objectivist position). We argue that Sarkissian et al.'s studies have some methodological limitations. In particular, participants may have assumed that the exotic or alien party misunderstood the harmful action, and this assumption, rather than a genuinely non-objectivist stance, may have contributed to the increase in non-objectivist responses. Study 1 replicated Sarkissian et al.'s results with additional follow-up measures probing participants' assumptions about how the exotic or alien party understood the harmful action, which supported our suspicion that their results are inconclusive and therefore do not constitute reliable evidence against the deflationary hypothesis. Studies 2 and 3 modified Sarkissian et al.'s design to provide a clear-cut and reliable test of the deflationary hypothesis. In Study 2, we addressed potential issues with their design, including those concerning participants' assumptions about how the exotic or alien party understood the harmful action. In Study 3, we manipulated the alien party's capacity to understand the harmful action. With these changes to the design, high rates of objectivism emerged, consistent with the deflationary hypothesis. Studies 4a and 4b targeted the deflationary hypothesis more precisely by manipulating perceptions of injustice to see the effect on objectivist responding and by probing the more specific notion of objectivism entailed by our hypothesis. The results fully supported the deflationary hypothesis.

## Introduction

The topic of this article is the implicit meta-ethics involved in moral beliefs—more exactly, involved in beliefs that it is wrong to cause harm to another person. Do ordinary people regard the accuracy of such beliefs as independent of any perspective on the matter, much like they regard the accuracy of the belief that the Moon is the Earth's only natural satellite (an objectivist position)? Or do they regard the accuracy of such beliefs as dependent on the perspective of the individual holding the belief (a subjectivist position), as dependent on the perspective of the social group holding the belief (a relativist position), or as baseless (a nihilist position)?

Traditionally, both psychologists and philosophers have argued that ordinary people regard moral beliefs, particularly those concerning harmful actions, as objectively correct (for *a priori* approaches, see Mackie, 1977; Blackburn, 1984; Brink, 1989; Smith, 1994; Shafer-Landau, 2003; for empirical approaches, see Nichols, 2004; Goodwin and Darley, 2008, 2012). And objectivism has often been taken as a hallmark feature of moral beliefs distinguishing them from other normative beliefs, such as the belief that it is wrong to eat a meal with one's fingers at the dinner table (see Turiel, 1983; Nucci, 2001; Skitka et al., 2005; Sousa and Piazza, 2014; Kumar, 2015) (For a general discussion, see Stanford, 2018).

However, there have been some dissenting voices claiming that ordinary people are not objectivists about the wrongness of harmful actions (e.g., Machery, 2018; Stich, 2019; Pölzler and Wright, 2020). In particular, Sarkissian et al. (2011) provided innovative evidence that arguably supports the idea that ordinary people are not objectivists—that they are relativists instead (see also Khoo and Knobe, 2018). Their results are especially relevant because, if reliable, they would show that people are not objectivists even in how they regard harmful actions that are deemed uncontroversially wrong.

The primary aim of this article is to defend a version of the traditional view on the topic of whether ordinary people are moral objectivists with respect to harm, using “harm” in the sense of pain/suffering or, more broadly, welfare reduction (cf. Feinberg, 1987; Bradley, 2012). We argue that harmful actions are regarded as objectively wrong if they are perceived to involve injustice. We claim that the studies by Sarkissian et al. (2011) have some methodological limitations and that, when the design of their studies is improved, high rates of objectivism are observed, consistent with our hypothesis. We also provide direct evidence that the perception of injustice is what drives people's objectivism concerning harmful actions, which directly confirms our hypothesis.

## Our Hypothesis and Previous Studies

In the present research, we tested the deflationary view of harm, which states: *if a harmful action is perceived to involve injustice, it is deemed objectively wrong*. This hypothesis has been elaborated and defended in a series of papers in the context of issues surrounding the Turiel tradition and the (im)morality of harm (Sousa, 2009; Sousa et al., 2009; Piazza et al., 2013, 2019; Sousa and Piazza, 2014; Piazza and Sousa, 2016; see also Berniunas et al., 2016).^1^

Along with the Turiel tradition and other research programs on the topic (see Turiel, 1983, p. 36; Nucci, 2001, p. 6; Skitka et al., 2005, p. 896; Sousa and Piazza, 2014, p. 5; Stanford, 2018, p. 37), the deflationary view claims that people's objectivism has a universalist scope—i.e., when people believe that a specific action is objectively wrong, they also believe that it is objectively wrong for any person (from any cultural background) to act in this specific way. There are two *potential* problems with this link between objectivism and universalism (for a more detailed discussion, see Goodwin and Darley, 2010; Goodwin, 2018).

The first problem is that there is no strict logical implication between the concepts of objectivism and universalism. Objectivism does not imply universalism. The belief *if one drops one's iPhone, it will fall down to the ground* is deemed objectively correct on Earth, but not universally correct (this belief applies to the Earth, but not to the Moon). Analogously, concerning wrongdoing, some Jews may believe that it is objectively wrong to eat pork, but that this belief does not apply universally (this belief applies to Jews, but not to Christians). Conversely, universalism does not imply objectivism. A subjectivist with regards to aesthetics may believe that it is wrong for all radio stations to play Britney Spears' music (i.e., universally wrong), but in terms of their own perspective on the matter: “When I claim that it is wrong for any radio station to play Britney Spears' music, this is solely based on my personal distaste for Britney Spears' music; I don't think there is any objective basis for this distaste.” Analogously, a moral subjectivist may believe that a harmful action is universally wrong, but in terms of their own perspective on the matter: “When I claim that it's wrong for anyone to do this harmful action, this is solely based on my personal opinion on the matter; I don't think there is any objective basis for this opinion.”

The second problem is related to the evidence coming from the moral-conventional task used by the Turiel tradition—in particular, from its *generalizability/universality probe* (see, e.g., Weston and Turiel, 1980; Wainryb, 1993; Yau and Smetana, 2003; see also the aforementioned references related to the deflationary view). With this probe, after judging that a harmful action is wrong in the context or culture of the participant, the participant is asked whether the action would still be wrong if carried out by a person in another context or culture where everyone thinks that the action is not wrong (including the person). If a participant says that the action is still wrong, this has been interpreted as evidence that the participant thinks that the action is universally and objectively wrong because the participant is saying that action is still wrong *even if* everyone in the other context or culture think that the action is not wrong. Nonetheless, this “still wrong” response does not exclude the possibility that the participant is a moral subjectivist as discussed above: the participant may take the correctness of their “still wrong” response simply as subjective. Thus, although the generalizability/universality probe is a valid measure of universalism, it is questionable whether it reliably measures objectivism.

The first problem is not in itself a real problem for it remains an important empirical question whether the objectivism supposedly connected to harmful wrongdoing has a restricted scope, has a universalist scope, or is underspecified in terms of scope, that is, whether people who believe that a harmful action is objectively wrong also believe that it is objectively wrong only for those within their social group to act this way, believe that it is objectively wrong for any person to act this way, or do not have any clear and stable belief in that respect. The deflationary hypothesis we are testing in this article claims that people's objectivism concerning harmful wrongdoing has a universalist scope *when it involves perceptions of injustice*. The second problem is indeed a real problem in that current evidence stemming from the moral-conventional task falls short of providing complete evidence for the deflationary hypothesis.

Independent of the Turiel tradition, there is an important methodology that probes objectivism less ambiguously: the *incompatible-beliefs paradigm*. In one version of the design, participants are asked to consider the incompatible beliefs of two appraisers—for instance, one appraiser believes that it is wrong to harm another person; the other believes that it is not wrong to harm another person. Then, participants are asked to judge whether the two beliefs are both correct (or whether only one of them is correct). If a participant accepts that only one of the beliefs is correct, this is taken as evidence that the participant's implicit meta-ethics is objectivist—presumably, the participant thinks that harming another person is objectively wrong.

Many results using this methodology (and ones like it) are indeed consistent with our hypothesis that people are objectivists concerning unjust harmful actions—e.g., cheating, stealing (for a review, see Goodwin, 2018). The great majority of adult participants accept that only one of two incompatible beliefs is correct with regards to such actions (see, e.g., Nichols, 2004; Goodwin and Darley, 2008, 2012). Furthermore, developmental work with children aged 5–13 years has shown that children are even more objectivist than adults in this respect (see, e.g., Wainryb et al., 1998, 2004). But while most current studies using the incompatible-beliefs paradigm provide clearer evidence about objectivism, they fall short of providing evidence on whether the objectivism at stake is one with a universalist scope, since the action evaluated is normally depicted in the context or society of the participant, rather than a context or society different from the participant's own. As a result, these studies too fall short of providing complete evidence for the deflationary hypothesis.

Be that as it may, Sarkissian et al. (2011) provided evidence that seems to undermine the deflationary hypothesis. They argued that existing findings using the incompatible-beliefs paradigm are somewhat misleading because the incompatible beliefs are attributed to appraisers from the same cultural background or psychological profile. These authors offer evidence that the rate of objectivist responding decreases significantly as the cultural or psychological distance between the opposing appraisers increases, even when the harmful actions being evaluated ostensibly involve injustice. In a series of innovative studies, dealing with normative beliefs concerning a harmful action like *Dylan buys an expensive new knife and tests its sharpness by randomly stabbing a passerby on the street*, Sarkissian et al. manipulated the cultural and psychological distance between two appraisers.^2^ In all conditions, one appraiser believes that the action is wrong, and a second appraiser believes that the action is not wrong (“is permissible”). In the first condition both appraisers are American; in a second condition one appraiser is American and the second appraiser is from an exotic culture living in the Amazon rainforest with quite different values than Americans; in a third condition one appraiser is American and the second appraiser is from an alien species with a psychology quite different from humans. Participants were asked to rate their agreement (on a seven-point scale, with 1 = strongly disagree and 7 = strongly agree) with the statement: “At least one of the judges must be mistaken.” Thus, a high level of agreement with the statement would indicate a predominantly objectivist position, whereas a low level of agreement would indicate a predominantly relativist position.

In the first study, Sarkissian et al. (2011) found that the level of agreement diminished significantly from the first to the third condition (means of 5.4, 4.4, and 3.2, respectively), showing that the greater the cultural and psychological distance between the appraisers the more participants held a relativist position. In other words, the greater this distance between the disagreeing parties, the more likely participants were to say that both are correct. In other studies, the authors replicated the same results to varying degrees, while making slight changes to the research design, including a change in which the action is described as occurring in a cultural context different from that of the participants of the study (i.e., the actor is an Algerian doing the stabbing in a foreign context, while the participants are Americans), thereby probing objectivism with a universalist scope.

It is important to note that two distinct features of these results may indicate a relativist stance. First, in absolute terms, when the two disagreeing parties were from radically different cultures or species, objectivist responses were no longer predominant (as shown by responses that were close to or below the midpoint, 4.00, of the agreement scale). Second, the degree of objectivist responding was significantly influenced by cultural or psychological distance, as revealed by the fact that the differences between the conditions of the studies were statistically significant. These two features are related, though logically independent. Sarkissian et al. interpreted both features as indicating a dormant relativistic tendency that had not been unearthed by previous studies: when people consider quite different appraisers disagreeing on ostensibly *unjust* harmful actions, most of them tend to be relativists. This therefore contrasts sharply with the conclusions drawn from earlier studies in which the opposing appraisers have similar cultural backgrounds or psychologies. And it apparently constitutes strong evidence against any hypothesis that people are generally objectivist concerning harmful actions, including the deflationary hypothesis, which emphasizes the role of perceptions of injustice in judgments of objective wrongdoing and understands objectivism more specifically as objectivism with a universalist scope.

We think Sarkissian et al. (2011) were right to identify limitations in the previous research, and that the overall strategy they used to remedy this was pertinent and original. Nonetheless, we do not think their results decisively resolve matters owing to potential issues concerning participants' interpretations of the disagreements. In their studies, the description of the stabbing action, reproduced above, does not completely specify all of the morally relevant aspects of the action (i.e., those aspects bearing on the perceived injustice of the act), which leaves open the possibility that the harmful action was understood as an instance of justifiable harm, rather than as involving injustice. This may not have affected participants' own understanding of the action, or their interpretation of the American appraisers' understanding of the action. But it may have led many participants to infer that the appraisers from a different culture or species had a very different understanding of the situation. For instance, participants may have inferred that, given their radically distinct background, the exotic and alien appraisers understood the harmful action as being one that was performed with the consent of the victim (e.g., as part of a cultural ritual), or because the victim was guilty of a crime, or for some other justifiable reason. In other words, the exotic and alien appraisers may be thought to have appraised fundamentally *different harmful actions*, actions that might be justified, even from the point of view of the participant.

Participants making such an inference might think the appraiser from a different culture or species is correct simply because they are judging a different action from the action the American appraiser has in mind. So, in evaluating whether the American appraiser or the exotic/alien appraiser is mistaken, these participants would not agree with the statement “at least one of the judges [appraisers] is mistaken.”^3^ Moreover, this pattern of relativist responding may have been further accentuated in the alien condition compared to the exotic-culture condition because the likelihood of participants inferring that the appraisers have different construals of the harmful action should increase as the cultural and psychological distance between the two appraisers increases.

If our point is relevant, Sarkissian et al.'s results are not conclusive with regards to the degree to which lay individuals are objectivist or non-objectivist regarding harmful actions involving injustice: One cannot determine whether the increase in relativist responding was due to some participants inferring that the two appraisers were judging different actions, or whether it was due to some participants truly thinking that the two appraisers were judging the same harmful action, while accepting that their incompatible normative beliefs about the action are equally correct (genuine relativism).

## Overview of Current Studies and Predictions

Study 1 replicates Sarkissian et al.'s design with follow-up measures to probe participants' assumptions about the appraisers' construal of the harmful actions. We predicted that participants would make different assumptions about the exotic and alien appraisers, showing that Sarkissian et al.'s results are inconclusive. If correct, this prediction undermines the main evidence against the deflationary hypothesis. Studies 2 and 3 modified aspects of Sarkissian et al.'s design in order to provide a more clear-cut and reliable test of the deflationary hypothesis. In Study 2, we addressed potential issues with their design, including those concerning the description of the morally relevant aspects of the harmful action. In Study 3, we manipulated the alien appraiser's capacity to understand the morally relevant aspects of the action. With these changes to the design, we predicted that high rates of objectivist responding would be observed, consistent with the deflationary hypothesis. Studies 4a and 4b manipulated the perception of injustice to examine its influence on the perception of objective wrongdoing with a universalist scope. We predicted that perceptions of injustice play a causal role in the perception of such objective wrongdoing. If correct, this prediction fully supports the deflationary hypothesis. In all studies, participants were U.S. residents recruited through Amazon's Mechanical Turk (www.mturk.com), and they were paid $0.40 for 5 min of their time. No participant responded to more than one study.

## Study 1

Study 1 consists of a direct replication of Sarkissian et al.'s design as discussed in the previous section—more specifically, of their study 4.^4^ However, to test our suspicion about the reason for their results, we added follow-up probes to assess whether participants assumed that the appraiser from a different culture or alien species had a different construal of the action scenarios. These follow-up probes allowed us to verify whether participants' apparently relativist responses are in fact driven by this type of assumption.

### Methods

#### Participants

The participants were 615 adults (255 male, 360 female; *M*_*age*_ = 38.25 years, *SD* = 12.36).

#### Design, Materials, and Procedures

The study had a 2 *act* (killing vs. stabbing) × 3 *appraiser pair* (American/American vs. American/Exotic vs. American/Alien) between-subjects factorial design. Participants were randomly assigned to read one of the following action scenarios:

**KILLING**. “Tom, a 40-years old American male, finds his youngest child extremely unattractive and therefore kills him.”
**STABBING**. “Curtis, an 18-year old College of Charleston student, buys an expensive new knife and tests its sharpness by randomly stabbing a passerby on the street.”

Within each scenario, participants were randomly assigned to one of three appraiser-pair conditions. In all conditions, one appraiser was described as an American student who thinks that the action is morally wrong. In all conditions, the second appraiser was described as an individual who thinks that the action is morally permissible. In the same appraiser condition, the second appraiser was also an American student described as follows:

“Imagine a student at Duke University named Sam. Sam's parents come from Poughkeepsie, NY. He enjoys going to the beach, watching college football, and hanging out with his friends. After graduation, Sam plans on traveling Europe for a while, then getting a job with a management consulting firm. Sam has very different moral views than most of his fellow students.”

In the exotic-culture and alien conditions, the second appraisers were described as members of the following groups, respectively:

“The Mamilons are an isolated tribe of people living in the Amazonian rainforests. Although the surrounding areas are now controlled by a technologically advanced civilization, the Mamilons have struggled to hold on to their traditional culture and rituals. The most important of these rituals is a coming-of-age ceremony that takes place when children reach the age of fourteen. At that age, children face a choice between leaving the tribe to join the surrounding civilization, or swearing a solemn vow to uphold the traditions of the Mamilonian people. Though some leave, many choose to stay and uphold these traditions, and undergo a period of intensive training, where they are brought to a sacred place and taught the ancient lore of the Mamilonian warrior culture.”

“Imagine a society of extraterrestrial beings called the Pentars. The Pentars have a very different psychology than us. They do not experience love, friendship, pleasure or pain. They do not pursue the sorts of goals that we do. Instead, their entire lives are organized around a single project—the effort to reshape every object they can find into perfect pentagons. They are extraordinarily rational and efficient in the way they work together in achieving this goal, and they can always count on each other's collaboration. However, if it turns out that they can best achieve the goal by killing other Pentars, they immediately go ahead and proceed with the killing (after which they reshape the dead Pentars into pentagons themselves).”

For the main dependent measure, participants were asked to rate their level of agreement or disagreement (on a seven-point scale, from disagree to agree) with a statement about the conflicting views of the two appraisers: “At least one of the judges must be mistaken.” Thus, a high level of agreement with the statement would indicate a predominantly objectivist position, whereas a low level would indicate a predominantly relativist position.

After completing the original experiment in one of its six conditions, participants were asked to justify their judgment and to answer three or four follow-up probes (depending on the condition). These follow-up probes assessed assumptions that participants made about how the second appraiser understood the situation. Before presenting these probes, participants were asked, “when you indicated whether at least someone must be mistaken, what did you assume about [second appraiser's] understanding of the situation?” The follow-up probes were as follows, depending on the conditions:

*Both Act conditions*
**WRONGDOING**. “I assumed that [second appraiser] understood that Americans consider it wrong to kill one's young child/to stab passers-by.”
**HARM AVERSION**. “I assumed that [second appraiser] understood that Americans don't like to be killed/that the passer-by did not want to be stabbed.”
*Killing conditions*
**HEALTH**. “I assumed that [second appraiser] understood that the child was completely healthy, and that the father killed the child simply because he did not like the appearance of the child.”
*Stabbing conditions*
**NO PROVOCATION**. “I assumed that [second appraiser] understood that the passerby did not do anything to provoke the stabbing.”
*Exotic-culture conditions*
**WARRIOR VALUES**. “I assumed that the Mamilon thought that the purpose of the killing was to demonstrate warrior values.”
*Alien conditions*
**PENTAGONS**. “I assumed that the Pentar thought that the purpose of the killing was to make perfect pentagons.”

Participants answered on a seven-point scale from “totally disagree” to “totally agree, with “neither agree nor disagree” as the midpoint. High agreement in most follow-up probes indicates that participants recognized that the second appraiser had an understanding of the situation congruent with the first appraiser. The only exceptions are the “warrior values” and “pentagons” probes. Agreement with these probes indicates recognition that the second appraiser had an understanding of the situation distinct from the first appraiser.

### Results

We excluded five participants—two because their justification indicated that they did not take the survey seriously (e.g., “I choose [sic.] randomly”), and three because they gave the same answer for all probes and their justification suggested that they did not read the survey (e.g., “I did my best”).

#### Replication of the Original Results

**Figure 1:**
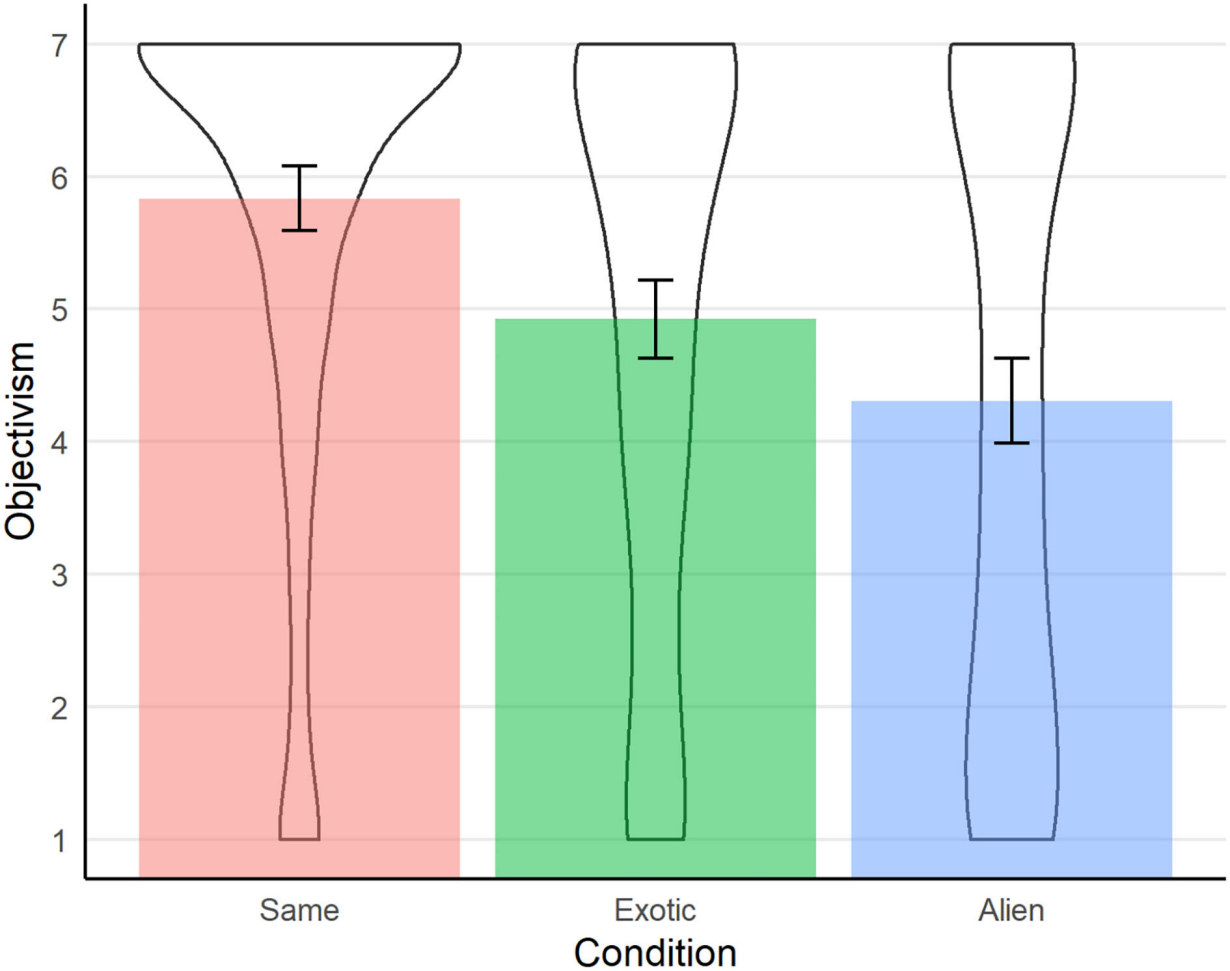
Agreement with the Objectivism probe for each appraiser condition, collapsed across scenario type (Study 1). Error bars represent 95% Confidence intervals, and violin plots represent densities of responses.

The results were very close to those originally found by Sarkissian et al.—the level of agreement with the objectivist probe diminished significantly from the first to the third of our appraiser conditions (see Figure 1). A 2 (act) × 3 (appraiser pair) between-subjects ANOVA revealed neither a main effect of act, *F*_(1, 604)_ = 1.52, *p* = 0.22, nor an interaction of act x appraiser, *F*_(2, 604)_ = 1.60, *p* = 0.20. There was however a significant main effect of appraiser, *F*_(2, 604)_ = 28.15, *p* < 0.001. Follow-up simple-effects tests showed that objectivist ratings were significantly lower in the Alien condition compared with both the Same condition (*M*_Alien =_4.3, *SD* = 2.3, vs. *M*_Same_ = 5.8, *SD* = 1.8), *d* = 0.74, *t*_(378.34)_ = 7.48, *p* < 0.0001, and the Exotic condition (*M*_Exotic_ = 4.9, *SD* = 2.1), *d* = 0.28, *t*_(398.2)_ = 2.81, *p* = 0.005. Objectivism ratings in the Exotic condition were also significantly lower than in the Same condition, *d* = 0.47, *t*_(391.05)_ = 4.70, *p* < 0.0001.

#### Assumptions of Misunderstanding Between Conditions

As predicted, participants thought the appraisers had different construals of the harmful actions. Follow-up probes tended to show the same decreasing pattern as the objectivism probe: Participants were most inclined to agree that the two appraisers had a shared understanding of the act in the Same condition, least inclined in the Alien condition, and intermediately inclined in the Exotic-culture condition (see Table 1; for the detailed statistics of all comparisons, see Appendix 1). This suggests that participants had not assumed that the Alien and the member of the Amazonian tribe understood crucial aspects of the situation, such as Americans' aversion to harm (M_*Alien*_ = 3.4, 95% CI [3.1, 3.7]; M_*Exotic*_ = 4.5, 95% CI [4.2, 4.8]) or the fact that Americans consider killing and stabbing to be wrong (M_*Alien*_ = 2.9, 95% CI [2.6, 3.2]; M_*Exotic*_ = 3.3, 95% CI [3.0, 3.5]).

**Table 1:** Descriptive statistics for all probes (Study 1).

	**Same**	**Exotic**	**Alien**
Objectivism	5.8^a^ (1.8)	4.9^b^ (2.1)	4.3^c^ (2.3)
Harm aversion	5.7^a^ (1.9)	4.5^b^ (2.1)	3.4^c^ (2.1)
Wrongdoing	5.4^a^ (2.0)	3.3^b^ (2.0)	2.9^b^ (2.0)
Health	5.5^a^ (2.1)	5.5^a^ (1.8)	4.7^b^ (2.1)
No provocation	6.0^a^ (1.6)	5.2^b^ (1.9)	4.6^c^ (2.2)
Warrior values	NA	4.1 (1.9)	NA
Pentagons	NA	NA	4.6 (2.2)

*Mean (SD) for all probes, by condition. Within each row, all means with different superscripts are significant at the 0.05 level (two-tailed, based on t-tests with Welch correction)*.

#### Predicting Objectivism Based on Assumptions of (Mis)Understanding

**Figure 2:**
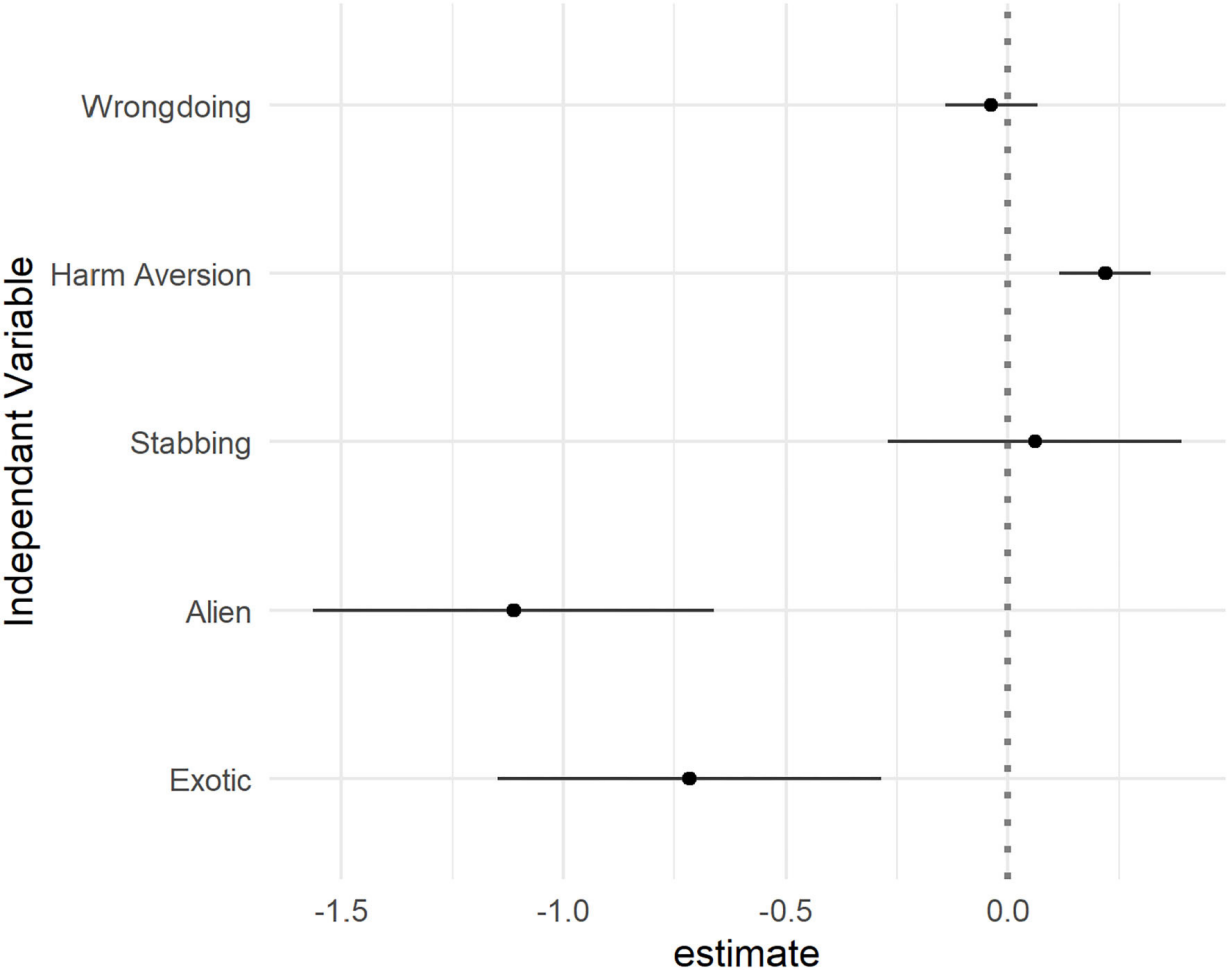
Linear regression predicting Objectivism based on Act type, Condition, Wrongdoing and Harm Aversion (Study 1). The “Killing” and “Same” conditions are used as baseline.

To further investigate the possibility that responses to the objectivist probe were based on participants' assumptions, we built a linear model to predict objectivism judgments based on the *Wrongdoing* and *Harm aversion* follow-up probes, controlling for act (Stabbing vs. Killing) and condition (using the Same condition as the baseline). We focus here on the *Wrongdoing* and *Harm aversion* probes because these were the only ones asked in all conditions. As can be seen in Figure 2 and Table 2, the assumption that the victim was averse to harm emerged as a significant predictor. Greater assumptions of understanding of *Harm aversion* positively predicted more objectivist answers. Controlling for *Harm aversion*, the *Wrongdoing* probe did not significantly predict objectivist responses. However, even when accounting for *Wrongdoing* and *Harm Aversion*, the Exotic and Alien conditions still had a significant impact on the objectivist probe. This could reflect participants' making additional assumptions about the appraiser's misunderstanding of the scenario that are separate from their assumptions about harm aversion (for instance, concerning the assumptions that we measured but did not include in the regression model because they were specific to only one type of action), a genuine effect of conditions on relativist stance, or any other methodological feature of Sarkissian et al.'s design that tended to promote relativist responses (see Studies 2–3 below).

**Table 2:** Linear regression predicting Objectivism based on Act type, Condition, Wrongdoing, and Harm Aversion (Study 1).

**Predictor**	***b***	**95% CI**	***t*(604)**	***p***
Intercept	4.76	[4.18, 5.34]	16.17	<0.001
Exotic	−0.72	[−1.15, −0.28]	−3.26	0.001
Alien	−1.11	[−1.56, −0.66]	−4.85	<0.001
Stabbing	0.06	[−0.27, 0.39]	0.36	0.723
Harm aversion	0.22	[0.12, 0.32]	4.17	<0.001
Wrongdoing	−0.04	[−0.14, 0.07]	−0.73	0.467

*The “Killing” and “Same” conditions are used as baseline*.

### Discussion

We replicated Sarkissian et al.' results. However, the decrease in objectivist responses across conditions was accompanied by a similar decrease in the assumption that the exotic and alien appraisers had a shared understanding of the harmful action in question. This suggests that, rather than reflecting a genuine relativist stance, relativist responses were based on the assumption that the exotic and alien appraisers were evaluating a different action. Of course, our results are correlational, and cannot be interpreted as indicating a causal link between misunderstanding and apparent relativist responses. We claim, however, that our results show that no straightforward interpretation of Sarkissian and colleagues' results is possible. As a consequence, interpreting their results as support for a kind of folk moral relativism concerning harmful actions is premature.

Study 1 provides correlational evidence that assumptions of misunderstanding underpin Sarkissian et al.'s (2011) results. In Study 2, we modified Sarkissian et al.'s (2011) design in several critical ways to avoid its potential limitations, including the likelihood that participants would view the two appraisers as having distinct construals of the action. Our aim here was not to locate the exact source of the problem with Sarkissian et al.'s results, but to provide a more clear-cut and reliable test of the deflationary hypothesis. We predicted that this more appropriate design would show that most people adhere to objectivism concerning harmful actions perceived to involve injustice, irrespective of appraiser condition.

## Study 2

### Methods

#### Participants

Participants were 244 adults (178 male, 66 female; *M*_*age*_ = 29.79 years, *SD* = 9.10).

#### Design, Materials, and Procedures

The study had a 2 *act* (killing vs. stabbing) × 3 *appraiser pair* (American culture/American culture vs. American culture/exotic culture vs. American culture/alien species) between-subjects factorial design.

Participants were randomly assigned to a killing or stabbing scenario:

**KILLING**. “An American father finds his *healthy* young child *physically* unattractive and for this reason kills him.”
**STABBING**. “An American student buys an expensive new knife and tests its sharpness by randomly stabbing *an innocent* person on the street *against their will*.”

The words in italics differ from Sarkissian et al.'s overall description of the scenarios. These additional details were added to reduce the possibility of participants inferring that the two appraisers construed the harmful action differently in morally relevant ways, i.e., in ways that pertain to the perceived injustice of the action. And they presumably make explicit what Sarkissian et al. wanted to convey with their description but left unspecified. In the stabbing scenario, “innocent” and “against their will” were introduced to eliminate a reading in which the harm was deserved or societally condoned (e.g., as part of a ritual), or in which the victim consented to the harm. In the killing scenario, “healthy” and “physically” were introduced to eliminate a reading in which the harm was a mercy killing (or served some other protective purpose, e.g., to prevent the spread of a fatal disease) and to emphasize that the motivation for the killing was merely aesthetic (hence, trivial and selfish).

Participants were then asked to imagine two individuals. The descriptions of these individuals were similar to the descriptions provided in Sarkissian et al.'s studies but simplified to include only the main elements that indicate cultural or psychological differences between the appraisers, thus streamlining the information for greater fluency in the context of an online study. In all three appraiser conditions, one individual was an American, and was described as follows:

“SAM is an American student who enjoys watching college football and hanging out with friends.”

In each condition, the second individual was either another American, someone from an exotic culture, or an alien:

“PETER is an American student who enjoys watching college football and hanging out with friends.”

“BAAKO is from an isolated tribe of people called the Mamilons, who live in the Amazon rainforests. The Mamilons have quite different values from those in our society—they cultivate a warrior tradition that values war and honor above all things.”

“VELVET is from an extraterrestrial alien species called the Pentars. The Pentars have a very different sort of psychology from human beings—they do not experience friendship, love, pleasure or pain.”

Participants were then asked to imagine that SAM and the second appraiser had the following thoughts about the harmful action:

SAM thinks: “What the American student/father did is wrong.”
PETER/BAAKO/VELVET thinks: “What the American student/father did is NOT wrong, even if Americans think it is wrong.”

Differing from Sarkissian et al., we added the clause “even if Americans think it is wrong” to the second appraiser's thought to avoid participants interpreting the thought *descriptively* in the exotic and alien conditions. Without this clause, participants may interpret BAAKO's or VELVET's thought as: “What the American student/father did is not wrong *for Americans*.” On this interpretation, participants would have reasoned that BAAKO or VELVET, not knowing much about American culture, mistook the harmful action as “not wrong” in American culture, and then simply expressed that misinterpretation. But this judgment only constitutes a description of what Americans think rather than a normative evaluation of the action. Consequently, if participants interpreted the situation in that way, they would deem BAAKO or VELVET incorrect just because they think that BAAKO and VELVET misrepresent what Americans think, and not because they themselves hold a genuinely objectivist position on the moral issue.

We used a more straightforward categorical measure that included a greater range of non-objectivist positions. Participants were prompted: “We are interested in your personal opinion about SAM and PETER's [BAAKO's/VELVET's] thoughts. Please select the option that best reflects your personal opinion.” Participants were then given three options. For example, in the alien-appraiser condition the options were:

(1) Only SAM's thought is correct.
(2) SAM's and VELVET's thoughts are both correct.
(3) There is no truth of the matter as to whose thought is correct.

Our first option can be plausibly interpreted as an objectivist response. If a participant believes that the action at stake is wrong objectively, she will accept that only Sam's thought is correct. Moreover, the reverse inference—that a “Correct” response indicates an objectivist position—is also highly plausible in the context of the other response options available in the task. Our second option is clearly a relativist option, while our third option could indicate other non-objectivist positions such as nihilism. We did not include the alternative objectivist option (e.g., “Only VELVET's thought is correct”) to simplify things, given that it is plausible to suppose that no one would seriously choose it. It is also important to note that Sarkissian et al.'s dependent measure does not unambiguously indicate an objectivist stance: a moral nihilist à* la* an error theorist (Mackie, 1977) could fully agree with the statement “at least one of the appraisers is mistaken.” Thus, one could argue that our three-option measure has enhanced validity, insofar as it allows for a greater range of plausible response options and given that past studies have generated similar overall results using both interval and categorical measures (e.g., Goodwin and Darley, 2008, 2012).

After selecting one of the three options, we introduced an open-ended question asking participants to justify their response, to further probe whether participants were interpreting the materials as intended and to identify patterns in participants' reasoning that are relevant to our hypothesis. Then, participants answered two follow-up dichotomous Yes-No measures to confirm that they understood the actions as involving injustice and basic rights violation.^5^ For the stabbing scenario, e.g., the probes read: “Has the American committed any injustice by stabbing this person?” “Has the American violated the rights of the person he stabbed?” Finally, participants answered some demographic questions and were debriefed, thanked, and paid. No other measures were collected.

### Results

#### Main Probe

The difference in the responses related to the Killing and Stabbing acts was not significant, χ^2^(2) = 0.78, *p* = 0.677, so we aggregated these responses. The percentages of responses to the main probe in each appraiser condition are presented in Table 3.

**Table 3:** Percentages of responses to the main probe by appraiser condition, collapsing across the two harmful actions, in Study 2.

	**Appraiser condition**		
	**Same**	**Exotic**	**Alien**
	**(*n =* 84)**	**(*n =* 80)**	**(*n =* 80)**
One correct	82%	74%	53%
Both correct	4%	15%	28%
No truth	14%	11%	19%

To determine whether there were significant differences between appraiser conditions, we conducted Chi-square tests with responses coded dichotomously—i.e., objectivist responses coded separately from non-objectivist responses (i.e., “Both correct” and “No truth” collapsed). Departing from Sarkissian et al.'s results, the difference in objectivist responses between the *same* and *exotic* appraiser conditions was not statistically significant, χ^2^(1) = 1.68, *p* = 0.194, φ = 0.10; however, consistent with Sarkissian et al.'s findings, the difference in objectivist responses between the *alien* and each of the other appraiser conditions was significant: *same*, χ^2^ (1) = 16.46, *p* < 0.001, φ = 0.32, and *exotic*, χ^2^ (1) = 7.76, *p* = 0.005, φ = 0.22. Goodness-of-fit Chi-square tests with responses coded dichotomously revealed that while objectivist responses in the *alien* appraiser condition were not significantly more prevalent than 50%, χ^2^ (1) = 0.20, *p* = 0.655, φ = 0.05, objectivist responses in the *same* and *exotic* appraiser conditions were the clear majority response (i.e., they were significantly more prevalent than 50%): χ^2^ (1) = 34.71, *p* < 0.001, φ = 0.64, and χ^2^ (1) = 18.05, *p* < 0.001, φ = 0.48, respectively. In other words, participants' responses were more divided within the alien condition, whereas their responses were predominantly objectivist within the same and exotic appraiser conditions.

#### Follow-Up Probes

Responses to the injustice and rights-violation probes were at ceiling level in all conditions (see Table 4), which confirms that, according to participants, the harmful actions at stake were consistently understood as instances of injustice and basic rights violation.

**Table 4:** Percentages of yes responses to the follow-up probes by appraiser condition, collapsing across the two harmful actions (stabbing and killing) in Study 2.

	**Appraiser condition**		
	**Same**	**Exotic**	**Alien**
Injustice	98%	100%	99%
Rights violation	98%	100%	99%

### Discussion

We discuss the Exotic and Alien conditions in turn, focusing on the two features that may indicate a relativist stance: whether, in absolute terms, people no longer tend to provide objectivist responses, and whether, in relative terms, there is a decrease in objective responses across these conditions.

We found that, with an improved design, the substantial majority of participants in the *exotic* condition offered an objectivist response, consistent with the deflationary hypothesis. Because we made various changes to Sarkissian et al.'s design, there is not a straightforward interpretation of which specific features caused the discrepancy between our results and theirs. We presume that the difference is in part due to the fact that our design made the morally relevant aspects of the harmful actions more explicit, thus mitigating assumptions that the two appraisers are conceptualizing different actions. But we cannot preclude the possibility that other differences may have contributed as well.

We also found that there was no significant difference in the level of objectivist responses exhibited between the *same* and *exotic* conditions. Still, there was a non-negligible difference between these conditions, and we do not deny that, even after having fully specified the harmful action in terms of its morally relevant aspects, there may be some measurable decline in objectivist responses from the *same* condition to the *exotic* condition. One possible explanation of this difference is that, in contrast to the same condition, the exotic condition is more likely to prompt a descriptive reading of the overall probe such that participants' responses reflect descriptive relativism rather than meta-ethical relativism. Consider this type of justification, associated with relativist responses, which was more frequent in the *exotic* than in the *same* condition:

“According to the cultural values held by each person, the thoughts are correct. Whether it is right or not is entirely different, but according to the values held by each person, it is consistent.” (*Exotic*/*Killing*)

“These two individuals have very different belief systems, and both believe their statements to be true.” (*Exotic*/*Stabbing*)

These participants state simply that the opposing thoughts of the appraisers are understandable from the perspective of the different backgrounds of the appraisers, which seems to indicate simply a descriptive stance on the matter. Such a descriptive stance does not imply anything about the correctness of the perspectives being described: it consists merely of a report of psychological or sociological facts. It is therefore entirely compatible with objectivism, whereas relativism is not. Moreover, a descriptive stance of this sort is necessary but not sufficient for relativism, since the latter consists of an evaluative claim about the validity of different perspectives—namely, that the perspectives are equally correct. Thus, rather than actively engaging with radically different perspectives in a way that leads to a genuinely relativist position, it may be that many participants respond to the *exotic* condition as if it called merely for a descriptive answer.^6^ If this is the case, it would have increased the number of apparently (but not genuinely) relativist responses in the *exotic* condition. Still, even in the face of this possibility, objectivist responses were predominant in both the *same* and *exotic* conditions.

In contrast, the results of the alien condition seem to provide strong evidence for folk relativism: not only were objectivist responses less frequent, but they were also significantly lower than the other conditions. However, we believe there are some problems with these results. Justifications evincing a descriptive reading of the probe were also present in the alien condition. More importantly, consider the following justifications:

“(…) Velvet is from a completely different world that is without emotion and love and therefore doesn't see anything wrong with what happened, and that makes her view correct in the eyes of her people.” (*Alien*/*Killing*)

“(…) From Velvet's perspective however, his alien species does not seem to have a conscience since they cannot experiences (sic.) those emotions and pleasure and pain. If one cannot experience these things then one (…) would not be able to differentiate right from wrong. (…)” (*Alien*/*Stabbing*)

These justifications suggest that many participants chose the non-objectivist option for reasons related to the description of the alien's bizarre psychology. The alien species, the Pentars, was described as not experiencing friendship, love, pleasure or pain. This obviously implies that Pentars lack certain experiential capacities. However, this could also imply that the alien lacked the ability to *understand* some of the morally relevant aspects of the action—e.g., the fact that humans generally do not want to be stabbed because it causes them pain, which is an aversive experience that most humans try to avoid. Accordingly, many participants may have been reluctant to deem VELVET incorrect simply because they thought that VELVET lacked the conceptual capacity to understand the morally relevant aspects of the action. If so, this would again mean that the two appraisers would have been rendering moral judgments on fundamentally different actions, and so the results from the *alien* condition would not necessarily constitute a challenge to the deflationary hypothesis. They would constitute a challenge, however, if high rates of non-objectivist responding remained within a context in which the alien species is described as capable of *understanding* the morally relevant features of a clearly unjust, harmful action.

To test this idea, in Study 3, we followed the overall design of Study 2, but focused exclusively on the alien appraiser condition and the stabbing scenario. We manipulated two different aspects of the alien species' bizarre psychology: their ability to *experience* various emotions, and their capacity to *understand* that humans do not like to be stabbed (i.e., the knowledge that the alien may have been thought to lack in Study 2). We predicted that when the psychology of the Pentars is described in a way that makes it clear that they can understand the morally relevant aspects of the harmful action (i.e., the aspects pertaining to the perceived injustice of the act), the majority of participants would select the objectivist response, consistent with the deflationary hypothesis.

## Study 3

### Methods

#### Participants

Participants were a sample of 238 adults (125 male, 118 female; *M*_*age*_ = 34.39 years, *SD* = 12.62).

#### Design, Materials, and Procedures

We used only the stabbing scenario in this study. After reading the scenario, participants were instructed to imagine two individuals “SAM” and “VELVET.” As in Study 2, SAM was described as “an ordinary American student who enjoys watching college football and hanging out with friends.” VELVET was described as “an extraterrestrial from an alien species called the Pentars.” Participants were randomly assigned to one of three conditions providing additional information about the Pentars' psychological characteristics, concerning the capacity to have *experiential states* (EXP) and the capacity to *understand* that humans normally find being stabbed undesirable because it causes pain (UND):

**EXP+UND**. The Pentars have the same sort of psychology as human beings—they EXPERIENCE friendship, love, pleasure and pain. They also UNDERSTAND that human beings find being stabbed undesirable because it causes them pain.
**NO EXP+UND**. The Pentars have a different sort of psychology from humans—they DO NOT EXPERIENCE friendship, love, pleasure and pain. However, they DO UNDERSTAND that human beings find being stabbed undesirable because it causes them pain.
**NO EXP+NO UND**. The Pentars have a very different sort of psychology from humans—they DO NOT EXPERIENCE friendship, love, pleasure and pain. They also DO NOT UNDERSTAND that human beings find being stabbed undesirable because it causes them pain.

We did not include the fourth possibility, in which VELVET would be capable of experiencing the relevant mental states (e.g., experiencing pain), but not capable of understanding them (e.g., understanding that pain is undesirable). This was done to simplify things and because we thought participants would find this condition unintelligible; that is, we thought that, for participants, it would be odd and difficult to imagine a being that could experience pain but that could not in some way understand that pain is undesirable.

Participants were then asked to imagine that SAM and VELVET have the following thoughts about the stabbing act:

SAM thinks: “What the American adult did is wrong.”
VELVET thinks: “What the American adult did is NOT wrong, even if Americans think it is wrong.” (for NO EXP+NO UND)
VELVET thinks: “What the American adult did is NOT wrong, even if Americans think it is wrong, and even if it causes the innocent person undesirable pain.” (for EXP+UND and NO EXP+UND)^7^

Participants were given the same three response options from Study 2, and the same justification probe. In addition to the injustice and rights-violation probes of Study 2, they were asked whether they personally thought the stabbing action was wrong. Before being debriefed, thanked, and paid, participants answered some demographic questions. No other measures were collected.

### Results

#### Main Probe

The percentage of responses to the main probe in each appraiser condition is presented in Table 5. Chi-square tests, with responses coded dichotomously as in Study 2, showed that the difference in objectivist responses between the EXP+UND and NO EXP+UND conditions was not statistically significant, χ^2^(1) = 0.35, *p* = 0.557, φ = 0.05, but the difference in objectivist responses between the NO EXP+NO UND condition and each of the other conditions was statistically significant: EXP+UND, χ^2^(1) = 11.56, *p* = 0.001, φ = 0.27, and NO EXP+UND, χ^2^(1) = 7.93, *p* = 0.005, φ = 0.22. Goodness-of-fit Chi-square tests with responses coded dichotomously (objectivist vs. non-objectivist) revealed that objectivist responses in the EXP+UND and NO EXP+UND conditions were significantly more prevalent than 50%: χ^2^(1) = 18.51, *p* < 0.001, φ = 0.49, and χ^2^(1) = 12.48, *p* < 0.001, φ = 0.40, respectively. Thus, when the alien species *understood* the morally relevant aspects of the harmful action, objectivist rates were predominant, consistent with our prediction. By contrast, objectivist responses in the NO EXP+NO UND condition did not differ significantly from 50%, χ^2^(1) = 0.11, *p* = 0.742, φ = 0.04, indicating that when the alien *did not understand* the morally relevant aspects of the harmful action, participants were no more likely than chance to select the objectivist response.

**Table 5:** Percentages of responses to the main probe by condition (Study 3).

	**Condition**		
	**EXP+UND (*n =* 78)**	**NO EXP+UND (*n =* 77)**	**NO EXP+NO UND (*n =* 83)**
One correct	74%	70%	48%
Both correct	6%	23%	34%
No truth	19%	7%	18%

#### Follow-Up Probes

Responses to the injustice and rights-violation probes confirmed that participants perceived the action at stake as involving injustice and basic rights violation in every condition (see Table 6). Additionally, virtually everyone personally thought the harmful action was wrong, independent of condition (see Table 6).

**Table 6:** Percentages of yes responses to the follow-up probes by condition (Study 3).

	**Condition**		
	**EXP+UND**	**NO EXP+UND**	**NO EXP+NO UND**
Wrongdoing	96%	95%	96%
Injustice	94%	96%	100%
Rights violation	100 %	94%	98

### Discussion

The results of Study 3 indicate that what likely drove down objectivist responses in the alien condition in our Study 2 (and arguably in Sarkissian et al.'s data) was not the fact that the alien holds a radically different moral perspective. Rather, the relevant difference rests in the alien appraiser's being unable to understand others' morally-relevant hedonic states—a specific psychological deficit that affects how the alien would interpret harmful events. This psychological deficit meant that, in the mind of many participants, the disagreement between the alien and the American could have arisen simply because they evaluated fundamentally different actions—in one case, an act that causes undesirable harm, and in another case, an act that does not cause undesirable harm—and not because the appraisers rendered fundamentally different moral judgments about the very same action. Accordingly, when the alien appraiser was described as properly understanding the morally relevant aspects of the harmful action (namely, that it caused undesirable suffering), the great majority of participants chose the objectivist option. Thus, by experimentally manipulating the absence vs. presence of understanding (i.e., one alien does not have the capacity to understand the nature of the action, while the other one does), Study 3 shows that the alien appraiser's understanding of the relevant act was critical to participants' judgments. In contrast, the alien's experience was not critical, since the alien's inability to experience hedonic states did not exert much influence on participants' judgments.

Study 1 undermined the evidence against the deflationary hypothesis. Studies 2–3 addressed potential issues with Sarkissian et al.'s design, thereby showing high objectivist responding, which is consistent with the deflationary hypothesis. Studies 4a and 4b extend our investigation in two ways to more precisely test the deflationary hypothesis. First, we directly manipulated the purported injustice of the harmful actions in question to examine the effect on judgments of objective wrongdoing. Second, we used a design that probes objectivism with a universalist scope—the more specific notion of objectivism entailed by the deflationary hypothesis. We predicted that a difference in the perception of injustice would make a positive difference in the judgment of objective wrongdoing with a universalist scope, which would fully support the deflationary hypothesis.

## Studies 4a and 4b

### Methods

#### Participants

In Study 4a, participants were 204 adults (110 male, 92 female, two missing data; *M*_*age*_ = 37.2 years, SD = 8.3). In Study 4b, participants were 254 adults (145 male, 109 female; *M*_*age*_ = 34.39 years, *SD* = 12.62).

#### Design, Materials, and Procedures

Study 4a had a 2 *act* (killing vs. stabbing) x 2 *context* (injustice vs. not-injustice) between-subjects factorial design. Study 4b had the same design but with an additional not-injustice condition. In both studies, the conditions started by describing a person (Baako) from an isolated Amazonian tribe of the people called the Mamilons, who have quite different values from the surrounding society. The remaining description was as follows for each of the conditions, the only difference between the common conditions of Studies 4a and 4b being that the harmful action was described in more detail in Study 4b (see content in brackets)^8^:

*Killing/Injustice (4a and 4b)*
Mamilons think that it is NOT wrong for a parent to kill a healthy child that they find physically unattractive, and it is common practice for Mamilon parents to do so. Baako finds his healthy young child physically unattractive and therefore he kills the child [by suffocation].
*Killing/Not-injustice (4a and 4b)*
Mamilons think that it is NOT wrong for a parent to kill an extremely sick child that is suffering from a deadly, contagious disease, and who is unlikely to improve at all or survive, and it is common practice for Mamilon parents to do so. Baako sees that his young child is sick with a deadly, contagious disease. The child is suffering and is unlikely to improve or survive, and therefore he kills the child [by suffocation].
*Stabbing/Injustice (4a and 4b)*
Mamilons think that it is NOT wrong for a person to test the sharpness of a knife by randomly stabbing an innocent person with it, and it is common practice for Mamilons to do so. Baako takes his new knife and tests its sharpness by randomly stabbing an innocent person against their will. [Baako, an elder, takes his knife and stabs the arm of a young, innocent Mamilon man against his will as a way of demonstrating his power.]
*Stabbing/Not-injustice (4a and 4b)*
Mamilons think that it is NOT wrong for young men to stab each other with sharp knives as a rite of passage into manhood, and it is common practice for young Mamilon men to do so. Baako takes his knife and stabs a young Mamilon man as a rite of passage into manhood. [Baako, an elder, takes his knife and stabs the arm of a young Mamilon man as a rite of passage into manhood.]
*Stabbing/Not-injustice (4b)*
Mamilons think that it is NOT wrong for Mamilon elders to stab young Mamilon men as a way of punishing them when they commit a seriously immoral act. It is common practice in Mamilon society for elders to do this, and everyone finds this practice acceptable. Young Mamilon know that they will be stabbed if they are caught committing a seriously immoral act. Baako, an elder, takes his knife and stabs the arm of a young Mamilon man as a punishment for his robbing the home of a fellow Mamilon.

After reading one of the above scenarios, participants were asked to imagine someone who is *not* a Mamilon (“Person A”). In Study 4a, Person A was described as having the following thought about Baako's action: “What Baako did is still wrong, even if the Mamilons do not think it is wrong.” In Study 4b, Person A was described as having the following thought: “Baako's action is inherently wrong, that is, it is wrong independent of any prevailing cultural norms.” In both studies, participants were then asked to select the option that best reflects their opinion: (1) Person A is correct; (2) Person A is incorrect. Person A's thoughts were supposed to describe an objectivist position concerning wrongdoing. Hence, if a participant chooses option “Person A is correct,” they are endorsing Person A's thoughts and therefore (indirectly) claiming that the harmful action is objectively wrong. Moreover, since the harmful action occurs in a different cultural context, not the context of the participant, this choice gives evidence not simply about the perception of objective wrongdoing but, more specifically, about the perception of objective wrongdoing with a universalist scope. Now, if a participant chooses the option “Person A is incorrect,” whether because they think that the action is not-objectively wrong, objectively not-wrong, or not-objectively not-wrong, the participant is denying that the harmful action is objectively wrong (Note that the deflationary hypothesis entails a prediction about *objective-wrongdoing*—see footnote 1).

It is worth pointing out that although the design for Studies 4a−4b departs from the typical incompatible-beliefs paradigm in terms of the type of measure probing objectivism, it still prompts participants to compare two disagreeing beliefs. The Mamilons (including Baako) think that the harmful action is not wrong, whereas Person A thinks that the harmful action is wrong. Since Person A is not a Mamilon, the scenario represents a moral disagreement among appraisers from different cultural backgrounds.

In both studies participants were asked an open-ended question to justify their response. To probe their perception of *injustice*, in Study 4a participants were presented with the question, “Has Baako committed any injustice by stabbing this person/killing his child?,” while in Study 4b they were presented with the question, “How much injustice has Baako caused by killing his sick/healthy child (stabbing the young/innocent man)?” A Yes-No option (1 = Injustice, 0 = No Injustice) followed the former question, while a 7-point scale (1 = No injustice at all, 4 = A moderate amount, 7 = A lot of injustice) followed the latter. In Study 4b, we also included a measure of perceived *selfishness* (“How selfish were Baako's actions?” 1 = Not at all selfish, 4 = Moderately selfish, 7 = Extremely selfish), which we take to be prototypically related to perceptions of injustice, as well as harm measures such as *pain* (“How much pain has Baako caused the sick/healthy child [the young/innocent man]?” 1 = No pain at all, 4 = A moderate amount, 7 = A lot of pain), *well-being reduction* (“How much has Baako negatively affected the well-being of the [individual of the scenario] by his actions?” 1 = Not at all, 4 = A moderate amount, 7 = A lot), and *societal health* (“By his actions, how much has Baako negatively affected the health of the Mamilon society?” 1 = Not at all, 4 = A moderate amount, 7 = A lot). Before being debriefed, thanked, and paid, participants answered some demographic questions.

### Results

Two participants (both from Study 4a) were eliminated from the samples because their justification clearly indicated that they chose the wrong option (e.g., they chose “Person A is incorrect” but then said “Killing is wrong, regardless of another's beliefs”).

#### Manipulation Checks

In terms of the injustice follow-up probes of each study, there was a significant difference between injustice and not-injustice conditions: for Study 4a, 90.5% vs. 51.5%, χ^2^(1) = 38.01, *p* < 0.001, ϕ = 0.43; for Study 4b, *M* = 5.83 vs. *M* = 3.15, *t*_(252)_ = 11.48, *p* < 0.001, *d* = 1.48. Thus, in both studies, our manipulation of perceived injustice was successful.

#### Injustice and Objective Wrongdoing

The percentages of objective-wrongdoing responses across conditions in each of the studies are presented in Tables 7, 8. In both studies, participants chose the objective-wrongdoing option significantly more often in the injustice conditions than in the contrasting conditions: in Study 4a, χ^2^(1) = 12.05, *p* < 0.01, ϕ = 0.34 for killing, and χ^2^(1) = 6.40, *p* < 0.05, ϕ = 0.25 for stabbing; in Study 4b, χ^2^(1) = 33.65, *p* < 0.001, ϕ = 0.57 for killing, χ^2^(1) = 15.49, *p* < 0.001, ϕ = 0.38 for the stabbing-ritual, and χ^2^(1) = 12.58, *p* < 0.001, ϕ = 0.35, for the stabbing-punishment. Collapsing across conditions, judgments of injustice were highly correlated with objectivist responding: for Study 4a, *r*_ϕ_(202) = 0.72, *p* < 0.001; for Study 4b, *r* (254) = 0.74, *p* < 0.001.

**Table 7:** Percentages of objective-wrongdoing responses by condition in Study 4a.

	**Injustice**	**Not-injustice**
Killing	88%	58%
	**Injustice**	**Ritual (Not-injustice)**
Stabbing	88%	66%

**Table 8:** Percentages of objective-wrongdoing responses by condition in Study 4b.

	**Injustice**	**Not-injustice**	
Killing	92%	37%	
	**Injustice**	**Ritual (Not-Injustice)**	**Punishment (Not-injustice)**
Stabbing	81%	43%	47%

#### Predicting Objective Wrongdoing

We ran binary logistic regressions with injustice, selfishness, pain, well-being reduction, and societal health as simultaneous predictors in each of three separate analyses for the data in Study 4b (see Table 9). These analyses aggregated within each action type, so they consisted of a single analysis of Killing (unjust and not unjust killing), and two separate analyses of Stabbing (1: unjust stabbing and ritual stabbing; 2: unjust stabbing and punitive stabbing). Perceptions of injustice were a significant predictor in all three analyses, and perceptions of selfishness, which we see as prototypically related to perceptions of injustice, were a significant predictor in two of the analyses. Measures of harm, however, were a poor predictor: the measure of individual harm (pain and well-being reduction measures) was not a significant predictor at all, while the measure of societal health, which may be considered a measure of utilitarian harm, was a significant predictor in only one analysis.

**Table 9:** Measures predicting objective-wrongdoing responses, separated by harm type (killing; ritual stabbing; punishment stabbing) pooling the injustice and not-injustice conditions in Study 4b.

**Harm type**	**Predictors**	**B**	**Wald(1)**	***P*-value**	**Tolerance**	**VIF**
**Killing (unjust** **and not unjust)**						
	Injustice	**0.49**	5.03	0.025	0.33	3.03
	Selfishness	**0.48**	4.53	0.033	0.31	3.22
	Pain	0.35	1.68	0.194	0.26	3.75
	Well-being	−0.04	0.01	0.897	0.43	2.32
	Societal health	0.28	1.78	1.181	0.65	1.54
**Stabbing 1 (unjust and ritual)**						
	Injustice	**0.76**	6.69	0.01	0.25	4.43
	Selfishness	**0.55**	4.08	0.043	0.25	3.91
	Pain	0.04	0.01	0.922	0.43	2.33
	Well-being	−0.37	0.98	0.320	0.23	4.29
	Societal health	**1.07**	8.40	0.001	0.40	2.49
**Stabbing 2 (unjust and punitive)**						
	Injustice	**1.13**	15.75	0.00	0.34	2.96
	Selfishness	0.01	0.01	0.944	0.51	1.95
	Pain	0.56	2.87	0.09	0.47	2.11
	Well-being	−0.20	0.42	0.514	0.29	3.43
	Societal health	0.31	1.62	0.203	0.40	2.48

*Bs in boldface are statistically significant. VIF, Variance Inflation Factor*.

### Discussion

There are two distinct aspects of the results of Studies 4a and 4b—one related to the injustice conditions, and another related to the contrast between the injustice and not-injustice conditions. The quite high rate of objective-wrongdoing responses in the injustice conditions not only bolsters the results of Studies 2 and 3, but also extends these results by showing that the objectivism endorsed by ordinary people has a universalist scope. It is also worth pointing out that, by evincing both objectivism and universalism, this aspect of the results speaks to two strands of the literature on folk objectivism, addressing the following concern raised by Stephen Stich, “Neither the Goodwin and Darley studies nor other studies exploring moral objectivism make any effort to show that the moral judgments they focus on would pass Turiel's test, or anything like it.” (Stich, 2018, p. 29). Moving to the other aspect, the much higher rate of objective-wrongdoing responses in the injustice conditions than in the not-injustice conditions shows that perceptions of injustice play an important causal role in people's perceptions of objective wrongdoing. In sum, the results fully corroborate the deflationary hypothesis. Still, one may raise some issues concerning these results.

First, concerning both aspects of our results, one may claim that the option “Person A is correct” in Study 4a does not necessarily indicate an objectivist position concerning wrongdoing because Person A's thought (“What Baako did is still wrong, even if the Mamilons do not think it is wrong”) does not unambiguously express an objectivist position. It is hard to see why a relativist or a nihilist would choose this option, instead of “Person A is incorrect.” Yet, it is indeed logically consistent that a participant would choose “Person A is correct” to mean “I agree with Person A: in my personal opinion what Baako did is still wrong, but I don't think there is any objective basis for this opinion,” thereby merely expressing a subjectivist position. In other words, it seems that we are back to the problem with the generalizability/universality probe of the moral-conventional task discussed in our review of the literature. However, although this is logically consistent, we believe most participants choosing “Person A is correct” in fact expressed an objectivist position. At least this interpretation was often conveyed by their justifications:

“I believe that there is such a thing as moral truths. (…)” (*Killing*)

“Person A is right, just because Baako doesn't have the moral intelligence to know that the stabbing is wrong does not make it okay.” (*Stabbing*)

More importantly, we replicated this result in Study 4b, in which the response probe differed from that in 4a, such that it articulated a more unequivocally objectivist position (“Baako's action is inherently wrong, that is, it is wrong independent of any prevailing cultural norms.”). Neither a relativist, a nihilist nor a subjectivist could choose the option “Person A is correct” here.

Second, concerning the second aspect of our results, one may claim that these studies are inconsequential for they just demonstrate that the deflationary hypothesis is trivially true—who would not predict more objective-wrongdoing responses in the injustice conditions than in the not-injustice conditions? We believe that this impression of triviality actually comes from the fact that our prediction corresponds to people's moral intuitions, in which case it provides further confirmation of our hypothesis. Moreover, consistent with the fundamental role of injustice hypothesized by the deflationary perspective, Study 4b showed that, concerning the contrasting conditions, measures of injustice (including measures of selfishness) were a good predictor of objective-wrongdoing responses, while measures of harm were a rather poor predictor. Therefore, we prefer to say that the deflationary hypothesis is precise rather than trivial (see the series of papers on the deflationary view mentioned earlier, in which we rebut similar critiques of triviality).

## General Discussion and Conclusion

The present studies tested the hypothesis that if a harmful action is perceived to involve injustice, it is deemed objectively wrong. Contrasting with previous studies, Sarkissian et al. (2011) provided evidence that people are not objectivists concerning harmful actions, even those actions that are ostensibly unjust. In Study 1, we replicated their results, but also showed that the interpretation of their results is compromised by the fact that participants may have assumed that the *exotic* and *alien* appraisers had a quite different understanding of the harmful action being evaluated. Moreover, with an improved design, Studies 2 and 3 indicated objectivist responses at levels on par with past results (e.g., Goodwin and Darley, 2008). Finally, Studies 4a and 4b provided direct evidence that perceptions of injustice influence objectivism with a universalist scope. Thus, overall, the results largely support the deflationary hypothesis.

We acknowledge that our studies have many limitations. For instance, our sample included only American MTurkers, and our scenarios involved only a few harmful actions. More evidence is needed to settle whether the deflationary hypothesis holds among more diverse samples and with a broader range of harmful actions. Also, because we were primarily interested in testing the deflationary hypothesis, rather than pinpointing the exact causal source of the difference between our results and Sarkissian et al.'s (including the results of our replication Study 1), further studies manipulating the various changes that we made in our studies are necessary to definitely establish this source. We would like to conclude by briefly discussing three other issues that deserve further clarification or refinement in the context of our perspective.

We interpret the results of Studies 1–3 as suggesting that, in the context of the evaluation of normative disagreements about an action, people's background assumptions about a shared or distinct understanding of the morally relevant aspects of a harmful action likely affect their second-order judgments about the disagreement (in particular, assumptions of *distinct* understanding seem to have led to reduced objectivist responding). This is a specific hypothesis we put forward to (at least partially) explain Sarkissian et al.'s results and should not be confused with the deflationary hypothesis. The deflationary hypothesis does not predict that all cases of normative disagreement that are perceived to involve a different understanding of the action would reduce objectivist responses. For instance, suppose that Liam, a pro-lifer, believes that abortion before 3 months of pregnancy is objectively wrong because it involves injustice. Now, suppose that Liam hears about the following disagreement: Leo (another pro-lifer) thinks that such abortion is wrong; Charles (a pro-choicer) thinks that such abortion is not wrong (To make an analogy with previous studies, think of Liam as a participant, Leo as the first appraiser, and Charles as the exotic appraiser). Finally, suppose that Liam knows that Charles thinks that such abortion is not wrong because of the belief that a fetus is not yet a person before 3 months of age (thus, Charles thinks that no injustice is at stake). The deflationary hypothesis predicts that Liam would judge that only Leo is correct even if Liam assumes that Charles has a distinct understanding of the morally relevant aspects of the harmful action (i.e., a false belief about when the fetus becomes a person). The point here is that the specific hypothesis we put forward to (at least partially) explain Sarkissian et al.'s results does not apply to this abortion example. What is the difference then between this abortion example and Sarkissian et al.'s studies? In our view, participants' assumptions about appraisers' misunderstanding in Sarkissian et al.'s studies involve (i) the attribution of false beliefs (i.e., the exotic appraiser held false beliefs about the morally relevant aspects of the harmful action), (ii) the presumption that these beliefs are easily rectifiable (i.e., if the exotic appraiser had less ambiguous information, he would readily rectify his beliefs and reach the same understanding of the morally relevant aspects of the action), and (iii) the presumption that if these beliefs were rectified there would not be a disagreement (i.e., the exotic appraiser would agree that the harmful action is wrong, which is the other side of our earlier point that participants thought that they could agree with the exotic appraiser if they had interpreted the situation as he did). In other words, participants are not assuming that there are fundamental disagreements in play. Nothing of the sort applies to the abortion example.

In studies of harmful actions involving injustice (including ours), there is always a relevant minority of respondents that provide genuine non-objectivist responses. Why do some people seem to adhere to non-objectivism concerning even these actions? According to Sousa and Piazza (2014) intuitions about the objective wrongness of injustice (e.g., someone harming another person with a callous or selfish disregard for the victim's basic interests) are generated by a specialized cognitive mechanism that evolved to maintain mutually beneficial interactions via the presumption of a reciprocal social contract obligating individuals to act in ways that do not infringe on the basic interests of those around them (see also Baumard et al., 2013). However, moral intuitions of this sort may be downplayed by reflective beliefs sanctioned in specific social environments or arrived at by other reasoning processes (Mercier and Sperber, 2017). Thus, some individuals end up with a reflective view of morality as fundamentally subjective or culturally relative (e.g., think of students of cultural anthropology) or as fundamentally void, reflecting a nihilist view (e.g., think of those who, after repeatedly witnessing the strategic usage of moral claims in the service of power, develop a completely cynical view of human nature). We surmise that this is the case with the minority of people who hold fast to non-objectivist positions concerning acts of injustice.^9^

We have not provided a precise characterization of meta-ethical objectivism in this article. One common characterization is as follows: a person assumes that *A is wrong* is objectively correct if and only if they accept (i) that *A is wrong* is true, (ii) that its truth is independent of any particular perspective on the matter (including the perspective of the person making the assumption), and (iii) that truth means correspondence with external facts. This characterization of objective correctness may suggest that ordinary people hold objective normative beliefs much like they hold factual beliefs—i.e., they accept some form of realism concerning normative properties (*A is wrong* is true because it corresponds to some external normative fact). However, this is not the only way of construing folk objectivism. Take the following disagreement: one person thinks that “2 + 2 = 4”; another thinks that “2 + 2 ≠ 4.” An objectivist concerning such a disagreement may think that only one person is correct without necessarily supposing that mathematical beliefs correspond to an external, platonic world of mathematical facts. This objectivist may simply have the intuition that it would be incoherent or absurd to deny that “2 + 2 = 4.” Similarly, when ordinary people indicate that harmful actions involving injustice are objectively wrong, they may not think that there is some moral “stuff” out there that somehow grounds their normative beliefs; rather, they may simply have a strong intuitive sense that it is incoherent or absurd to think that harmful actions involving injustice are not wrong, given the inferences delivered by the specialized cognitive system that evolved to maintain mutually beneficial interactions (Sousa and Piazza, 2014). In sum, our results do not speak to point (iii) above. People's objectivist intuitions about moral wrongdoing might be realist, but they might also be “coherentist”, in which case their appeals to meta-ethical realism may be a way of justifying to others (and of making sense to themselves) their strong first-order intuitions. This issue warrants further research.

Regardless of how ordinary people construe the objectivity of moral beliefs, the present research contributes to an important debate in the literature. It highlights several methodological concerns to consider when probing objectivism. It provides new evidence supporting the idea that ordinary people are objectivists about harmful actions involving injustice. It helps illuminate why some previous research has come to the opposite conclusion. And it further elaborates the deflationary hypothesis about the (im)morality of harm.

## Data Availability Statement

The full data for our five studies can be found on the OSF page of this project: https://osf.io/xde4u.

## Ethics Statement

The studies were reviewed and approved by The Ethics Committee of the School of History, Anthropology, Philosophy and Politics at Queen's University, Belfast (Study 1) and The Institutional Review Board at The University of Pennsylvania (Studies 2, 3, 4a, and 4b). The participants provided their written informed consent to participate in all studies.

## Author Contributions

GG, PS, AA, and JP designed Study 1, while PS, GG, and JP designed Studies 2, 3, 4a, and 4b. JP collected the data of Studies 2, 3, 4a, and 4b, while AA collected the data of Study 1. All authors participated in the analysis of the data, although AA was primarily responsible for reporting the statistics of Study 1. PS led the writing of the paper but with substantial contributions from GG, AA, and JP.

## Conflict of Interest

The authors declare that the research was conducted in the absence of any commercial or financial relationships that could be construed as a potential conflict of interest.

## Publisher's Note

All claims expressed in this article are solely those of the authors and do not necessarily represent those of their affiliated organizations, or those of the publisher, the editors and the reviewers. Any product that may be evaluated in this article, or claim that may be made by its manufacturer, is not guaranteed or endorsed by the publisher.

## Footnotes

^1^We have delineated both a *simple* deflationary view, on which the perception of injustice is a sufficient condition for a judgment of objective wrongness, and a *fully* deflationary view, on which the perception of injustice is both a necessary and sufficient condition for such a judgment. More formally, where “*I*” and “*OW*” stand for perceived injustice and perceived objective wrongdoing and “x” for a variable ranging over actions perceived to cause harm, the simple and fully deflationary hypotheses are ∀x(*I*x → *OW*x) and ∀x(*I*x ↔ *OW*x), respectively (*cf*. Sousa and Piazza, 2014). Note that in this paper we focus on the sufficiency hypothesis. Note also that our hypothesis is primarily about the concepts and inferences involved in people's interpretation of harmful actions and only secondarily about how participants in specific studies respond to questions probing whether harmful actions are objectively wrong.

^2^In most of their studies, the action was described implicitly or explicitly as occurring in the cultural context of the participants (i.e., Dylan is supposed to be an American doing the stabbing in the context of the United States).

^3^Note that for this pattern of responses to occur, the above inference does not have to involve a very precise interpretation of the exotic/alien appraiser's construal of the harmful action—some participants may have thought, e.g., that the exotic appraiser interpreted the stabbing as a consensual rite of passage that men went through; but some participants may have thought simply that, given that the action is somewhat vague, it is probably the case that the exotic appraiser is thinking of a different action, one in which presumably the harm is justified from the point of view of the exotic appraiser (e.g., “the harm was deserved or consented to by the victim”).

^4^More precisely, we replicated the *Local agent transgression* condition of Study 4. The *Local agent transgression* condition only differs from the other vignettes used by the authors in that it clearly specifies that the actors (Stabber or Killer) are American. We wish to thank the authors, who provided us with the original materials. The only difference between their design and our replication is that while in their design the act variable (killing vs. stabbing) was within-subjects in our replication it was between-subjects.

^5^We see the concept of injustice as closely tied to the concept of basic rights violations, a point that we did not have space to discuss here (see Sousa and Piazza, 2014).

^6^For a detailed discussion of descriptive readings of normative measures as a potential confound, see Sousa (2009) and Sousa and Piazza (2014).

^7^We introduced the additional clause “even if it causes the …” in the conditions involving understanding to emphasize that VELVET could understand that pain is undesirable.

^8^Note that, in comparison with the exotic and alien conditions of Studies 1–3, the conditions here provide more specific contextual information on why exactly the harmful action at stake is deemed not wrong, which arguably is an improvement in terms of design. Such details likely prevent some participants from making inferences about the reasons behind the distant appraiser's judgment that the harmful action is not wrong and get them to focus more directly on the question probing objectivism. By providing less information on that matter, Studies 1, 2, and 3 may have led participants to wonder why exactly the exotic or alien appraisers judged that the harmful action at stake is not wrong, potentially causing some of the participants to miss the point of the objectivism probe—e.g., participants may have interpreted the question as asking whether the appraiser indeed thinks that the action is not wrong. Consequently, after thinking about the context, participants may then have answered descriptively like “Yes, the appraiser does think that the harmful action is not wrong” (see discussion of descriptive relativism in Study 2).

^9^Some results in the literature (see, e.g., Goodwin and Darley, 2008, 2012; Wright et al., 2013; Beebe and Sackris, 2016) show that people's objectivism is moderated by some variables (e.g., religiosity, age, or the level of public controversy concerning the action in dispute), an issue that, for lack of space, we could not discuss in this article. We reckon at least part of this moderation is to be explained along the lines of our discussion here.

## Appendix

**Table A1:** Comparisons between the different conditions of Study 1.

**Probe**	**Same, *N =* 207**	**Alien, *N =* 202**	**Welch *t*-test**
Objectivism	5.8 (1.8)	4.3 (2.3)	t = 7.5, *p < *0.001
Harm aversion	5.7 (1.9)	3.4 (2.1)	t = 12, *p < *0.001
Wrongdoing	5.4 (2.0)	2.9 (2.0)	t = 12, *p < *0.001
Health	5.5 (2.1)	4.7 (2.1)	t = 2.5, *p =* 0.012
No provocation	6.0 (1.6)	4.6 (2.2)	t = 5.3, *p < *0.001
**Probe**	**Exotic**, ***N****=*****201**	**Alien**, ***N****=*****202**	**Welch** ***t*** **-test**
Objectivism	4.9 (2.1)	4.3 (2.3)	t = 2.8, *p =* 0.005
Harm aversion	4.5 (2.1)	3.4 (2.1)	t = 5.1, *p < *0.001
Wrongdoing	3.3 (2.0)	2.9 (2.0)	t = 1.8, *p =* 0.070
Health	5.5 (1.8)	4.7 (2.1)	t = 2.8, *p =* 0.005
No provocation	5.2 (1.9)	4.6 (2.2)	t = 2.3, *p =* 0.022
**Probe**	**Same**, ***N****=*****207**	**Exotic**, ***N****=*****201**	**Welch** ***t*** **-test**
Objectivism	5.8 (1.8)	4.9 (2.1)	t = 4.7, *p < *0.001
Harm aversion	5.7 (1.9)	4.5 (2.1)	t = 6.2, *p < *0.001
Wrongdoing	5.4 (2.0)	3.3 (2.0)	t = 10, *p < *0.001
Health	5.5 (2.1)	5.5 (1.8)	t = −0.13, *p =* 0.9
No provocation	6.0 (1.6)	5.2 (1.9)	t = 3.0, *p =* 0.003
